# How to Run a Laundry

**Published:** 1916-05-06

**Authors:** 


### HOW TO RUN A LAUNDRY.

### II. The Week's Work.

The management of the laundry and the weekly routine must be arranged to facilitate the observance of two rules. The first, never allow the patients' washing to be mixed with that of the staff; the second, nothing must be left of the week's work to stand over till the next week. Everything must be returned, and the laundry be perfectly empty, clean and tidy, by 1 o'clock on Saturday.

In order that the maids may be able to start straightaway at 7 A.M. on Monday it is necessary that the soiled-linen cupboards in the wards should be emptied on Saturday mornings; their contents are sorted, bagged, and sent to the laundry in the course of the morning and remain there unopened till Monday. The washing that has accumulated in the wards over the week-end and is sorted on Monday mornings reaches the laundry about 9.30 A.M. A great deal of trouble and time will be saved in the laundry if the sorting and counting of the soiled linen are carefully done in the wards. Ward sisters should see that each kind of article is done up into separate bundles or given bags to themselves. For instance, sheets will probably fill two bags, blankets one; another bag will contain several bundles of towels, pillow-cases, and bed-jackets; while a fifth will contain only nightgowns.

When the laundry is opened on Monday morning the bags are unfastened, and those containing sheets are dealt with first. The head-laundress books the numbers sent from each ward, and they are sorted into two classes, the cleaner ones being put into one washer, and dirtier ones into another. Foul linen, which has first been brushed and partially disinfected in the wards, is, of course, dealt with separately. It is important in filling the machines to leave plenty of room for the sheets, etc., to be well tossed. Rinsing is also a matter of great importance; four waters should be used if the clothes are to be a good colour. The amount of soap required varies according to its quality and the hardness of the water—as much must be used as will form a good lather. Soda is added if the clothes are very dirty.

Waterproof overalls and rubber boots should be provided for the two maids who are at work in the wash-house, for the process of lifting wet clothes out of the machines is a sloppy business, even if the clothes are left for ten minutes after the rinsing-water has run out to allow as much water as possible to drain away before handling them. When the wet clothes are taken out of the machines they are placed in the trolleys and wheeled to the hydro-extractor. This machine is like a cauldron with holes in it, and great care is needed in putting in the clothes, or the immense speed with which it revolves will cause the more tearable portions of garments to become entangled, and the pulling which is difficult to avoid in unpacking the machine results in considerable damage being done. In order to avoid this each article should be carefully rolled into a bundle, with sleeves, bibs, embroidery, and strings innermost, and packed into the machine as tightly as possible. At the end of ten minutes' centrifugalising in this machine the contents are ready for ironing, and are again placed in a trolley and conveyed to the ironing-room. Sheets are now passed through the calender. If they are moderately narrow one maid can feed the machine while two fold and place them on the rails of the drying-closet horses, and by the time the maids go to their lunch they will leave a goodly pile of clean sheets aired and ready for use. The ward washing is finished on Tuesday, so far as the wash-house is concerned. All sheets and pillow-cases are returned to the wards on Tuesday; other articles are not returned till Friday. On Wednesday the staff's washing is done while the ward ironing is proceeded with. On Thursday sheets and pillow-cases are again sent from the wards, and are returned with the other ward washing on Friday evening. Saturday morning is spent by the senior maids in sorting and sending off the staff's washing and by the juniors in scrubbing and cleaning and tidying up. The machines are covered up and the laundry left empty and spotless, ready for next week's washing. Theatre towels, overalls, etc., also babies' garments are washed daily, with the exception of Saturdays and Sundays. Scarlet jackets and other woollen garments should be washed by hand, and the former must never be mixed with other coloured garments. A golden rule in the laundry is to wash each class of garment separately as far as possible, and never allow dirty clothes to be washed with cleaner ones.

It is a bad arrangement to send probationers on errands to the laundry. In order to avoid friction, all complaints, whether made by the ward sister or any other person, should be made to the assistant matron, who will refer them to the matron if necessary. A written order, signed by the ward sister, should accompany a request for linen before the usual time of delivery, and the matron will discourage the practice as far as possible by arranging for such orders to be sent to the office when the laundry is closed on Saturday. Printed laundry books should be provided for each nurse and member of the staff, as well as for each ward, out-patients, theatre, etc., and such books are, of course, carefully checked by the head-laundress. It is a good plan to have a few simple rules as to the number of articles allowed, and the time and day the washing should be ready, to which is added a warning that all losses must be reported within a week, printed inside the cover of each book.

A plan of a hospital laundry will be found in The Hospital for August 22, 1914, *The Institutional Worker Supplement*, page 1.

